# Regional age-related changes in neuronal nitric oxide synthase (nNOS), messenger RNA levels and activity in SAMP8 brain

**DOI:** 10.1186/1471-2202-7-81

**Published:** 2006-12-21

**Authors:** Damien Colas, Abdallah Gharib, Laurent Bezin, Anne Morales, Gérard Guidon, Raymond Cespuglio, Nicole Sarda

**Affiliations:** 1 EA 3734, Faculty of Medecine, Claude Bernard University, 69373 Lyon, France; 2 UMR 5123, Bâtiment Dubois, 43 boulevard du 11 novembre 1918, 69622 Villeurbanne, France; 3 UMR 628, Faculty of Medecine, Claude Bernard University, 69373 Lyon, France

## Abstract

**Background:**

Nitric oxide (NO) is a multifunctional molecule synthesized by three isozymes of the NO synthase (NOSs) acting as a messenger/modulator and/or a potential neurotoxin. In rodents, the role of NOSs in sleep processes and throughout aging is now well established. For example, sleep parameters are highly deteriorated in senescence accelerated-prone 8 (SAMP8) mice, a useful animal model to study aging or age-associated disorders, while the inducible form of NOS (iNOS) is down-regulated within the cortex and the sleep-structures of the brainstem. Evidence is now increasing for a role of iNOS and resulting oxidative stress but not for the constitutive expressed isozyme (nNOS). To better understand the role of nNOS in the behavioural impairments observed in SAMP8 versus SAMR1 (control) animals, we evaluated age-related variations occurring in the nNOS expression and activity and nitrites/nitrates (NOx^-^) levels, in three brain areas (n = 7 animals in each group). Calibrated reverse transcriptase (RT) and real-time polymerase chain reaction (PCR) and biochemical procedures were used.

**Results:**

We found that the levels of nNOS mRNA decreased in the cortex and the hippocampus of 8- vs 2-month-old animals followed by an increase in 12-vs 8-month-old animals in both strains. In the brainstem, levels of nNOS mRNA decreased in an age-dependent manner in SAMP8, but not in SAMR1. Regional age-related changes were also observed in nNOS activity. Moreover, nNOS activity in hippocampus was found lower in 8-month-old SAMP8 than in SAMR1, while in the cortex and the brainstem, nNOS activities increased at 8 months and afterward decreased with age in SAMP8 and SAMR1. NOx^- ^levels showed profiles similar to nNOS activities in the cortex and the brainstem but were undetectable in the hippocampus of SAMP8 and SAMR1. Finally, NOx^- ^levels were higher in the cortex of 8 month-old SAMP8 than in age-matched SAMR1.

**Conclusion:**

Concomitant variations occurring in NO levels derived from nNOS and iNOS at an early age constitute a major factor of risk for sleep and/or memory impairments in SAMP8.

## Background

Aging is defined by an increase in the probability of death over time associated with characteristic changes in phenotype [1]. Sleep alteration is a common phenomenon observed in humans and animals during aging and is often associated with impaired learning and memory [2]. The oxidative stress resulting from the loss of control of reactive oxygen species (ROS) and the production of nitric oxide (NO), when produced in excess and not balanced by antioxidants, represent an attractive process responsible, at least partially, for the above impairments during aging or related disorders such as Alzheimer disease (AD) [3]. Our recent data obtained in aged rats [4] and in senescence accelerated prone 8 mice (SAMP8) [5], a model of early onset of human diseases [6], support such an hypothesis.

NO is an intracellular multifunctional molecule involved in both physiological and neurodegenerative processes of the CNS. The dynamic of the NO release taking place in the thalamus and the cortex [7,8] and/or locally in the basal forebrain of the rat [9] has been pointed as determinant in the modulation/regulation of the sleep-wake states and the maintenance of the brain homeostasis. In using pharmacological approaches (intraperitoneal, subcutaneous or local injections of NO donors or inhibitors) or KO mice, it has been, indeed, established that modulations of nitric oxide synthases (NOS), affect the sleep architecture in rodents. From these studies, NO derived from the neuronal NOS isoform (nNOS) is confirmed as a powerful sleep-facilitating agent whereas, NO produced by the inducible NOS (iNOS) plays a major role in triggering and maintenance of rapid-eye movement sleep (REM) during aging. Thus, nNOS and iNOS have complementary roles in the regulation of the sleep-wake cycle [9-14].

However, NO can also be neurotoxic and implicated in neurodegenerative diseases according to its free radical properties [3]. Previous studies have shown that NOSs expression is altered in AD. For example, in humans, neurons in the entorhinal cortex and hippocampus, that are vulnerable to neurodegeneration, express low levels of nNOS while iNOS plays a role in the formation of plaques and neurofibrillary tangles [15,16]. A similar pattern of NO dysfunction is observed in the senescence-accelerated mouse (SAM), a model of accelerated aging. In this way, various findings have suggested that accumulation of the damages exerted by NO-derived from iNOS together with the alterations resulting from glutamate signaling and cholinergic deficits lead to the degeneration of neurons and glia in brain. These impairments are also accompanied by learning and memory deficits occurring before the median age of survival of SAMP8 mouse [17,18]. By contrast to iNOS, it is still unclear whether nNOS is causally linked with the hippocampal dysfunction, the behavioral deficits and the neuronal death. The results obtained vary from an increase to a significant reduction in the nNOS activity and the protein content in the cerebral structures of the aged rat or SAM mouse [19-22].

In the present study, we evaluated the age-related variations occurring in nNOS expression and activity, nitrites/nitrates levels (NOx^-^) in the frontal cortex, the hippocampus and the brainstem of SAMP8 or senescence accelerated-resistant (SAMR1, controls aging normally) mice. Together with previous data [13], results obtained point out that SAMP8 unlike SAMR1 are exposed at an early stage to alterations of the NO derived from nNOS and iNOS. Such alterations might be causally linked to the senescence-related impairments and the degeneration observed in the brain of SAMP8.

## Results

### Age-dependent mRNA expression

**Figure 1:**
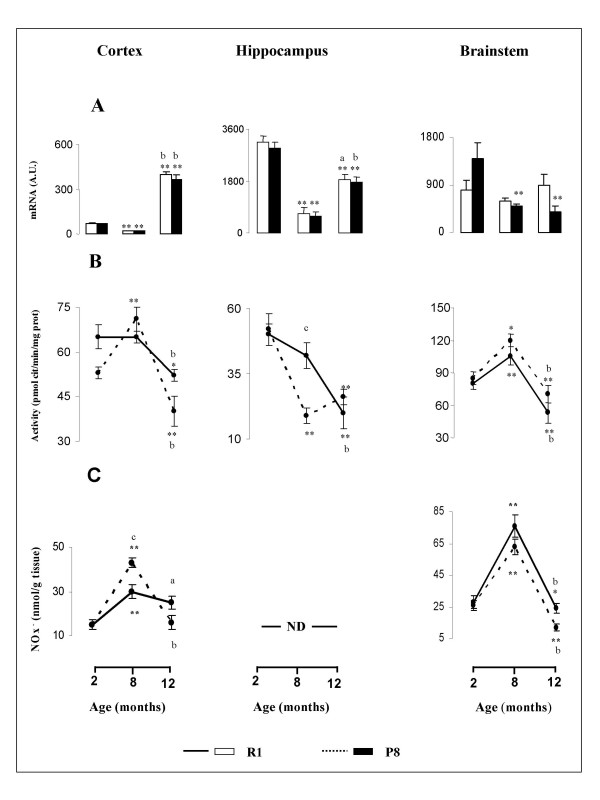
**Biochemical characterization of nNOS during aging in the cortex, the hippocampus and the brainstem of SAMR1 and SAMP8**. nNOS mRNA levels in arbitrary units (A.U.) (A), nNOS enzymatic activity in pmol of citrulline per minute per mg of protein (B) and Nitrites and nitrates (NOx) levels in nmol per mg of tissue (C) were detected in SAMR1 (solid line and open box) and SAMP8 (dotted line and dark box). Data are mean ± S.E.M) (n = 7 mice in each group). Statistics: significantly different from 2 months *P < 0.05, ** P < 0.01; or from 8 months ^a^P < 0.05, ^b^P < 0.01; significantly different from strain ^c^P < 0.01.

Data from RT-PCR are shown in Fig. 1A. From 2 to 8 months of age, a significant decrease (P < 0.01) in nNOS mRNA level was seen in the three brain areas of SAMP8 but only in the cortex and the hippocampus of SAMR1. From 8 to 12 months, nNOS mRNA level was reduced (P < 0.01) in the brainstem of SAMP8 while a significant increase (P < 0.01) takes place in the cortex and hippocampus of SAMP8 and SAMR1. When comparisons were made between 2-vs 12-months of age, results indicate that nNOS mRNA level was reduced (P < 0.01) in the hippocampus but increased (P < 0.01) in the cortex of SAMP8 and SAMR1. In 12 month-old animals, nNOS mRNA level was significantly lower (P < 0.01) in the hippocampus but significantly higher (P < 0.01) in the cortex than in 2 month-old ones. By contrast, nNOS mRNA level was lower (P < 0.01) in the brainstem at 12-vs 2 months of age, only in SAMP8.

### Age-dependent nNOS activity and NO^-^x production

The profiles of age-related changes occurring in nNOS activity are given in Fig. 1B. In SAMP8, nNOS activity increased (P < 0.01) in the cortex and the brainstem from 2 to 8 months, and then decreased (P < 0.01) in 12-vs 2 or 8 month-old mice. By contrast, nNOS activity markedly decreased (P < 0.01) in the hippocampus at 8-vs 2 months of age and remained low with age. In SAMR1, nNOS activity did not change significantly in the cortex and the brainstem from 2 to 8 months but afterward, it decreased (P < 0.01) with age. So, nNOS was lower (P < 0.01) in12-vs 2 or 8 month-old SAMR1.

The variations occurring in NOx^- ^levels are shown in Fig. 1C. NOx^- ^levels were higher (P < 0.01) in the cortex and the brainstem of 8-vs 2 month-old SAMP8 and SAMR1, the maximal increase (P < 0.01) occurred in the cortex of SAMP8 and was followed by a decrease with age. So, NOx^- ^levels were lower in the cortex (P < 0.01) and the brainstem (P < 0.01) of 12-vs 8 month-old SAMP8 and SAMR1. Finally, NOx^- ^levels were higher (P < 0.01) in the cortex of 8 month-old SAMP8 than in age-matched SAMR1. In the hippocampus, NOx^- ^were undetectable (limit of detection: 1 pmol/mg of tissue) in both SAMP8 and SAMR1.

## Discussion

In the central nervous system NO is produced by NOSs. Three different isozymes have been identified and nNOS accounts for the majority of the physiological actions of NO [20]. It is well documented that nNOS and iNOS have complementary roles in sleep regulation, learning and memory [9,14,15,21] and neuronal death in neurodegenerative disorders [3,23]. Previously, we demonstrated that the loss of iNOS activation is responsible to sleep impairments (i.e low quantities of REM sleep) at an early age in SAMP8 [13]. To extend our previous hypothesis on the putative role of NO in the accelerated brain aging of SAMP8, in this study, we examined the age-related changes occurring in the nNOS expression and activity and NO^-^x levels within the brainstem sleep-structure, the cortex and the hippocampus of SAMP8 and compared them to age-matched SAMR1. The main findings obtained indicate the existence of biphasic changes in NO metabolism: 1- a down-regulation of nNOS mRNA at 8 months of age followed by a slight up-regulation with age except within the brainstem; 2- an increase in nNOS activity and NOx^- ^levels, followed by a decrease with age, in the cortex and the brainstem in SAMP8 and SAMR1. By contrast, our data show a marked decrease in nNOS activity in the hippocampus at 8 months of age, i.e. well before aging, only in SAMP8. These results agree with previous data and suggest that the down-regulation of nNOS activity at an early age is causally linked with accelerated brain aging in SAMP8. The fact that nNOS activity remains constant with age reinforces such an evidence [15,19-22]. However, our present data also indicate that: -nNOS mRNA levels are not well correlated with activity values; NO^-^x levels are undetectable in whole hippocampi. This latter point, suggests that a more appropriate dissection is necessary to consider the part played by NO in the dorsal hippocampus and mainly in which regards the spatial information processing. Other studies conducted in human and rodents agree with our present findings. In this sense, it has been reported that: 1- different regions behave differently during aging and/or degeneration [16]; 2- nNOS expression and synthesis can be regulated by Ca^2+^fluxes independently from nNOS activity especially in the hippocampus [24]; (c) there is a post-transcriptional regulation of nNOS expression in the brain of SAM [17].

In the same way, the neurotoxicity of NO has been now extensively investigated and it appears that the NO production confers resistance to glutamate excitotoxicity [25]. Behind this observation, the precise mechanisms involved still remain unclear. Recently, it has been reported that neurons of the entorhinal cortex and the hippocampus, expressing low levels of nNOS, are highly vulnerable to degeneration, [15,16]. Other studies have shown that nNOS activity increases in several brain regions of aged SAMP8, SAMP10 and rats [17,22,26] suggesting that the increase production in NO can lead to an increased release of glutamate in the brain with aging [25]. On the other hand, studies have reported that iNOS expression is altered in AD and is implicated in the formation of plaques and fibrillary tangles [27]. Several mechanisms are involved in the neurotoxic effects exerted by NO. This free radical can combine with superoxide to form peroxynitrite, another highly destructive radical moiety. The resultant ROS can induce oxidative stress and cause lipid peroxydation, functional alterations in proteins and DNA and an eventual neuronal death. A dysfunction of Ca^2+ ^homeostasis supports also the link between NO and AD since Ca^2+ ^is the primary regulator of nNOS expression [3]. Furthermore, a relation between amyloid-β (Aβ) on NO production has been discussed. Recently, it has been reported the contribution of Aβ the cognitive decline in the aged SAMP8, since a reduction of Aβ by antibody or antisense oligonucleotides, reduces oxidative stress and improves learning and memory [6]. Our present results extend the observation that nNOS may be involved in the oxidative stress observed in the brain of SAMP8 and highlight the possibility that reduced NO/nNOS synthesis within the hippocampus at an early age may be a marker of vulnerability. These points need further investigations based on behavioral tests coupled with histochemical and/or biochemical analyses. Concerning the brainstem-sleep structures, our data support the idea that the sleep deterioration observed in the SAMP8, may result from complementary iNOS and nNOS alterations i.e a down-regulation of iNOS [13] concomitant with an activation of nNOS activity at an early age. By contrast, evidence supports the hypothesis that the NO synthesis derived from iNOS activity and its release, taking place within the brainstem, play a determinant role in the regulation of the sleep-wake states in SAMR1 aging normally [13,19].

Finally, NO emerges as a bi-functional regulator of apoptosis but, in the brain, it exerts only a pro-apoptotic effect during aging. Although the mechanisms behind its action have been investigated, the downstream mediators in NO-induced apoptosis are not yet well defined. Nonetheless, it has been reported that a high NO production, by up-regulation of nNOS expression promotes Bcl-2-linked apoptosis in the cortex of SAMP10 and causes an accelerated aging of the brain [22]. However, there is no significant difference in the expression of pro-apoptotic protein p53 content values between SAMP10 and SAMR1 [22] as observed in SAMP8 (unpublished results). More recently, it has been suggested that other factors such as low glial cell line-derived neurotrophic factor (GDNF) as well as other neurotrophines such as neurotrophine-3 and nerve growth factor [28] are involved in hippocampal dysfunction and neuronal death (including apoptosis in the hippocampus) in SAMP8. Regarding other structures, several indices of oxidative stress as well as alpha synuclein and hyperphosphorylated tau protein could rely to the senescence-related impairments and neurodegeneration in SAMP8 [29].

## Conclusion

Many alterations of the gene expression and protein abnormalities, such as deposition of Aβ-peptide, with relevance to age-related cognitive decline have been found in the brain of SAMP8. Interestingly, all studies support the hypothesis that oxidative stress leads to cognitive dysfunction. Our present findings together with previous data [13] demonstrate that SAMP8 unlike SAMR1 are really exposed at an early stage of their life to alterations of the NO metabolism. Therefore, we propose that concomitant variations occurring in NO levels derived from nNOS and iNOS at an early age constitute a major factor of risk for sleep and/or memory impairments.

## Methods

### Animals

Male SAMP8 and SAMR1 derived from AKR/J strain were obtained from INRA-Dijon, (colony providing from Kyoto University (Japan). Immediately after arrival (3 weeks of age), animals were maintained in individual cages and placed in a sound-isolated room (22°C ± 1°C; light-dark schedule 12 h- 12 h, light-on at 05. 00 h a.m., food and water *ad libitum*). The animals use and procedures were conducted according to the EEC Directive (86/609/EEC). Efforts were made to minimize the animal number, pain and discomfort. Two series of animals (7 animals/group) were sacrificed between 10.00 and 12.00 h, a convenient interval time for nNOS activity did not exhibit diurnal variations. Their brains were quickly removed and dissected on ice according to Glowinski and Iversen's method [30], i.e. frontal cortex, whole hippocampus and brainstem were separated from the whole brain and retained for assays. All samples were frozen on dry ice and stored at -80°C until RNA extraction and biochemical measurements.

### Reverse transcriptase polymerase chain reaction (RT-PCR)

The main procedure was similar to our previous description [13]. Briefly, total RNA was isolated from all tissues using the Trizol reagent (Invitrogen, USA) according to the manufacturer's instructions. To remove any genomic DNA contamination, total RNA was treated with RNAse-free DNAse I treatment (Qiagen) and purified using RNAeasy mini columns (Qiagen). Total RNA concentration was carefully determined by measuring A260 in a total volume of 50 μL using a Biophotometer (Eppendorf) and disposable RNAse-free UVettes. A260/A280 was also determined for each sample, and the values ranged between 1.81 and 1.90, without any difference between groups of mice. Using oligod(T)_15_, first-strand cDNAs were obtained from the reverse transcription of mRNAs contained in 500 ng of respective total RNAs (from the three brain regions in both SAMP8 and SAMR1 (n = 7 mice) (126 individual cDNA syntheses) in the presence of 80 pg of a synthetic external non-homologous poly(A) standard RNA (SmRNA) to normalize the reverse transcription reaction of mRNA of biological samples according to previous protocol [31]. All cDNA amplifications were realized on 1/20^th ^of the volume of the reverse transcription (corresponding to 25 ng of the initial total RNA solution). Real-time PCR amplification of nNOS-cDNAs was performed on a LightCycler (Roche Diagnostics) and was used to determine the initial concentration of templates in samples from a calibration curve (scale of nNOS-cDNA concentration = 6 logs; PCR efficiency = 1.89 with crossing points ranging from 16.51–34.71). The crossing points of all samples were within the range of the calibration curve. Quantitect SYBR Green PCR kit (Qiagen) was used, with the following parameters: 95°C for 15 min for denaturation, 50 cycles at 94°C for 15 sec, primer-dependent annealing temperature for 20 sec, 60°C for 20 sec and a final extension step at 72°C for 15 sec. Primers used (forward: 5'-ACC CAA CGT CAT TTC TGT CC-3'; reverse: 5'-AAG GTG GTC TCC AGG TGT GT-3') for the amplification of a 297 pb fragment of the mouse nNOS cDNA were chosen according to GenBank access number NM_008712. These primers yielded no PCR product on all samples tested in this study when PCR amplification were performed directly on 25 ng of total RNA (prior to reverse transcription of mRNAs), excluding the possibilities 1) that primer-dimer amplication occurred during PCR, and 2) that PCR products resulted from genomic DNA contamination. Using this primer pair, PCR amplification provided a single DNA product which was identified by a uniform melting temperature (85.44°C). The identity of the PCR product was confirmed by sequencing (MWG Biotech). Results obtained for nNOS mRNA were normalized against the SmRNA [31]. Results are expressed in arbitrary units (A.U)

### Measurements of nNOS activity and NOx^- ^metabolites

In a second series, brains were dissected on ice and structures retained (see above) were homogenised and treated as previously described [12]. Homogenates were centrifuged at 10 000 g for 45 min at 4°C to prepare crude cytosol supernatants. Aliquots were then stored at -30°C. Total NOS activity in the supernatant fluid was determined by measuring the conversion of [^3^H]L-arginine to [^3^H]L-citrulline in presence of calcium and calmodulin as we have fully previously described [12,13]. All assays were performed in duplicate. To determine the contribution of iNOS (Ca^2+^-independent) to total NOS activity, calcium was replaced with EGTA as previously described [13] and constitutive NOS activity [cNOS activity = nNOS + endothelial nitric oxide synthase (eNOS) activities] was computed by subtracting it from the total NOS activity. The preferential localization of eNOS to particulate fractions, presumably plasma membrane rather than supernatant fractions minimizes its contribution even if a substantial level of particulate eNOS activity in the brain regions particularly in hippocampus pyramidal cells cannot be excluded [32]. nNOS (90% of cNOS) was expressed in pmol cit/min/mg prot. An aliquot of the supernatant fraction was used to determine the levels of NOx^- ^by Griess reaction as previously described [12]. Each aliquot was deproteinised with NaOH and incubated for 30 min at 30°C in presence of Cu-coated Cd in order to reduce NO_3_^- ^to NO_2_^-^. Results are expressed as nmol/g tissue. Protein concentration in supernatant fractions was determined using Bradford's method [33].

### Statistic analysis

All results are expressed as means ± standard errors of means (S.E.M). Comparisons between groups were made using the one-way analysis of variance ANOVA followed by Bonferroni's test (multiple comparison). Differences were considered significant at P < 0.05.

## Abbreviations

The abbreviations used are: Aβ, amyloid beta; AD Alzheimer's disease; Ca^2+^, calcium; REM, rapid eye movement sleep; ROS, reactive oxygen species; SAM, senescence-accelerated mouse; SAMP8, senescence-accelerated prone mouse; SAMR1, senescence-resistant prone mouse.

## Authors' contributions

DC carried out animal work and performed all the biochemical studies. AG participated in the design of the study and adapted the determinations of nNOS and NO^-^x to the protocol. LB conceived the design of RT-PCR measurements. AM participated in the design of RT-PCR. GG helped in the graphics. RC helped in the last version of the manuscript. NS coordinated and supervised the development of the study, was responsible for the project giving economical support and helped in the redaction of the manuscript. All authors read and approve the final manuscript.
